# Skeleton-Structure WS_2_@CNT Thin-Film Hybrid Electrodes for High-Performance Quasi-Solid-State Flexible Supercapacitors

**DOI:** 10.3389/fchem.2020.00442

**Published:** 2020-06-12

**Authors:** Xinyu Yang, Jiahui Li, Chengyi Hou, Qinghong Zhang, Yaogang Li, Hongzhi Wang

**Affiliations:** ^1^State Key Laboratory for Modification of Chemical Fibers and Polymer Materials, College of Materials Science and Engineering, Donghua University, Shanghai, China; ^2^School of Materials Science and Engineering, Nanyang Technological University, Singapore, Singapore; ^3^Engineering Research Center of Advanced Glasses Manufacturing Technology, Ministry of Education, Donghua University, Shanghai, China

**Keywords:** tungsten disulfide, carbon nanotubes, hybrid film, quasi-solid-state, flexible supercapacitors

## Abstract

The purpose of this work is to explore the application prospects of WS_2_ as an active material in flexible electrodes. Since WS_2_ has similar disadvantages as other two-dimensional layered materials, such as easily stacking, it is essential to develop a three-dimensional structure for its assembly in terms of electrochemical performance. In addition, the low conductivity of WS_2_ limits its application as flexible electrode material. In order to solve these problems, carbon nanotubes (CNTs) are introduced to improve the conductivity of hybrid WS_2_ materials and to construct a skeleton structure during WS_2_ assembly. Compared with pure CNTs and WS_2_, the WS_2_@CNT thin-film hybrid with a unique skeleton structure has a high specific area capacitance that reaches a maximum of 752.53 mF/cm^2^ at a scan rate 20 mV/s. Meanwhile, this hybrid electrode material shows good stability, with only 1.28% loss of its capacitance over 10,000 cycles. In order to prove its feasibility for practical application, a quasi-solid-state flexible supercapacitor is assembled, and its electrochemical characteristics (the specific area capacitance is 574.65 mF/cm^2^) and bendability (under bending to 135° 10, 000 times, 23.12% loss at a scan rate of 100 mV/s) are further investigated and prove its potential in this field.

## Introduction

In the few past decades, with the rapid development of flexible electronic devices (Wang et al., 2015; Choi et al., 2016) and wearable electronic devices (An et al., 2017), flexible and deformable energy storage devices, which are the real bottleneck hindering flexible electronics from becoming ubiquitous in practical products (Zhang et al., 2018c), have gradually become a research hotspot. Supercapacitors (SCs) combining the properties of a broader temperature window, high power density, and simple structures that can often be safely charged or discharged in seconds with extremely long cycle life are among the most promising candidates for flexible and energy storage devices (Shao et al., 2015; Myung et al., 2019). Normally, there are two kinds of storage mechanism for SCs: electrical double-layer capacitance (EDLC) (Li et al., 2018) and pseudocapacitance (Brousse et al., 2015). Carbon-based materials, which act as active materials to contribute EDLC as electrode materials, have large surface areas, controllable pore size, and high chemical stability, but their single micropore structure affects their capacitive performance (Borenstein et al., 2017; Zhang et al., 2018a,d). For example, graphene, one of the most common electrode materials for SCs, has the advantages of large specific surface area and high electrical conductivity (Jadhav et al., 2019). However, graphene tends to aggregate due to its soft skeleton, and the actual specific surface area is much lower than the theoretical value, resulting in relatively low capacitance performance (Han et al., 2014; Iakunkov et al., 2019).

Two-dimensional (2D) transition-metal dichalcogenides (TMDs), such as WS_2_, MoS_2_, and VS_2_ (Khalil et al., 2016), have attracted tremendous interest owing to their similarities with graphene nanostructures and their unique chemical and physical properties (Ramakrishna Matte et al., 2010; Tang et al., 2015; Choudhary et al., 2018; Zhang et al., 2018b; Lin et al., 2019). The intrinsically layered structure of 2D TMDs enables the facile incorporation of ions between 2D layers separated by subnanometer physical gaps (Choudhary et al., 2016), which favors fast ionic adsorption/transport through them. Most 2D TMDs do not present sufficiently high electrical conductivities because they have a 2H phase crystal structure (the coordination of its metal atoms is trigonal prismatic) (Acerce et al., 2015) with a monolayer bandgap (Susarla et al., 2017), unlike zero-bandgap graphene (Tolar, 2019), which hampers the direct applications of 2D TMDs for SCs. Due to their poor electronic conductivity, the utilization of this electrode has been largely constrained. Therefore, changing from semiconducting 2H-phase to metallic 1T-phase is a way to improve the electronic conductivity of 2D TMDs. As promising pseudocapacitive materials, 1T-WS_2_ and 1T-MoS_2_ possess ideal capacitance behavior, abundant raw sources, and environmental friendliness. The 1T-MoS_2_ phase is 10^7^ times more conductive than the semiconducting 2H-MoS_2_, and the specific capacitance of the metallic 1T-WS_2_ electrode is 12 times higher than that of semiconducting 2H-WS_2_ (Acerce et al., 2015; Khalil et al., 2016). Although the electrochemical performance of chemically exfoliated 2D TMDs (Eda et al., 2011) has been improved, there is still the problem of easy stacking of 2D layers, which limits its application as a supercapacitor electrode in practice. In order to meet the requirements for practical application, improving the conductivity of 2D TMDs is one of the problems to be solved.

Therefore, it is advisable to consider inserting other low-dimensional nanomaterials between layers to alleviate the stacking situation. Here, based on 1T-WS_2_, carbon nanotubes (CNTs) were introduced, which served as a stack-proof skeleton to support the nanosheet layers of WS_2_. Benefitting from the regulation of composite nanostructures, the constructed hydrophilic free-standing nanocomposite electrodes achieve high specific surface area and high specific capacitance. In addition to playing the role of a stack-proof skeleton, the introduction of CNTs also solves the defect of the poor conductivity of WS_2_. At the same time, the synergetic effect also plays an important role in boosting the performance of the hydrophilic self-supporting nanocomposite WS_2_@CNT hybrid films. Quasi-solid-state flexible SCs are also assembled and investigated, and we find that they exhibit excellent electrochemical performances and cyclic stability, which indicates that the nanocomposite film electrodes exhibit prospective value concerning high-density energy storage in practical application.

## Experiment

### Preparation of 1T-WS_2_

1T-WS_2_ nanosheets were prepared by lithium intercalation. First, 500 mg bulk WS_2_ powder was dispersed in 0.5 mL n-butyl lithium hexane solution and slowly stirred for 5 days under the protection of argon for the intercalation reaction. Then, n-hexane was added for dilution and washing through a centrifugation process at 8,000 rpm for 10 min. Centrifugation was repeated three to five times until the residual organic lithium compounds had been removed. The obtained Li-intercalated WS_2_ composite was then dispersed in deionized water for ultrasound for 2 h, followed by dialysis for 24 h for purification to remove Li ions completely. Finally, the purified suspension was centrifuged at a speed of 8,000 rpm for 15 min to remove the unstripped WS_2_, and the stripped 1T-WS_2_ nanosheets were obtained.

### Dispersion of CNTs

First, 100 mg CNTs were dispersed in 200 mL deionized water, and 300 mg sodium dodecyl benzene sulfonate (SDBS) was slowly added as a surfactant while stirring until completely dissolved. A moderate amount of concentrated hydrochloric acid (HCl) was then added to the fume hood, and the pH was adjusted to 3–4. The obtained dispersion was ultrasonically treated in the ultrasonic cleaning machine for 30 min, and the cell grinder was used for ultrasonic treatment for 2 h; then, the well-dispersed 0.5 mol/mL CNT dispersion was prepared.

### Preparation of WS_2_@CNT Hybrid Film

WS_2_@CNT hybrid films were prepared by a self-assembly method via vacuum filtration. As-prepared WS_2_ dispersions and CNT dispersions were mixed in different proportions (mass ratio of CNTs:WS_2_=1:6~5:1) to prepare two groups of WS_2_@CNT dispersions of the same volume. The mixture was treated with ultrasound for 5 min, and the mixed solution was filtered with a circulating water vacuum filter so that WS_2_ nanosheets and CNTs self-assembled into a macroscopic hybrid film. When the water on the surface of the film had drained, the film was removed from the suction filter and was placed in liquid nitrogen. It was then frozen for half an hour to freeze the water inside the film that had not completely drained during filtration, and then it was put in a freeze dryer for 3 h to directly evaporate the ice crystals to form the WS_2_@CNT hybrid film. The key to the formation of the unique skeleton structure is to preserve the longitudinal arrangement of the carbon tubes in the direction of the drawn-out water flow. Another group of films for comparison were filtered at room temperature until completely dry. The microstructure and electrochemical properties of the two groups of films prepared by different methods will be compared later, so as to illustrate the importance of the stack-proof skeleton structure for high-performance thin-film hybrid electrodes.

### Assembly of a Quasi-Solid-State Flexible SC

As-prepared WS_2_@CNT hybrid film was cut into a size of 1.0 cm × 1.0 cm as the electrode. 1 M PVA/H_2_SO_4_ was used as gel electrolyte. Conductive silver paste and copper tape were connected to the edge of each electrode as the pole ear. Kapton was used to seal the polar ear, leaving an effective area of 0.25 cm^2^ for the actual electrochemical charge and discharge.

### Characterization

The phase structure of the samples was characterized by X-ray diffraction (XRD, pressure pipe: 40 kV, current: 30 mA, CuK α, λ = 1.54056 Å) with 2θ from 10° to 70°. Raman spectra were obtained using a Renishaw in plus laser Raman spectrometer with λexc = 532 nm. Surface analysis of the sample was studied by X-ray photoelectron spectroscopy (XPS, AXIS–ultra spectrometer, Kratos Co.). The microscopic morphology of the sample was characterized by Hitachi s-4800 field emission scanning electron microscopy (FE-SEM) and Talos F200S high-resolution scanning transmission electron microscopy (HR-TEM). The electrochemical properties of the film electrode were tested by a traditional three-electrode system: 1 M H_2_SO_4_ as electrolyte, Ag/AgCl_2_ as the reference electrode, Pt as the counter electrode, and the as-prepared WS_2_@CNT film cut into strips 0.5 cm wide as the working electrode, without a current collector and with an actual work area of 0.25 cm^2^. Cyclic voltammetry (CV) measurements were at a voltage window from −0.2 to 0.7 V and with a scanning speed from 20 to 1000 mV/s. Galvanostatic charge/discharge (GCD) curves were evaluated with different current densities. In addition, the cyclic stability of the electrode was also tested. Then, a two-electrode system was used for the test: the WS_2_ @ CNTs film was cut into two circles with equal area (*r* = 0.15 cm, *S* = 0.07 cm^2^) as working electrodes, 1 M H_2_SO_4_ was used as electrolyte, and a Swagelok cell was assembled for testing. CV measurements were made in a voltage window from 0 to 1.0 V and with scan rates from 20 to 1000 mV/s. GCD curves were evaluated at different current densities. The cyclic stability of the electrode was also tested (scan rate of 100 mV/s, cycling 10,000 times). The electrochemical performance of the quasi-solid flexible SCs was tested by using a two-electrode system. Two as-prepared samples with the same area were used as working electrodes, 1.0 M PVA/H_2_SO_4_ was used as gel electrolyte, and the actual charging and discharging area was 0.25 cm^2^. Specific capacitance is one of the important parameters of the electrochemical properties of an electrode and can be calculated according to the following equation:

(1) $$\begin{array}{l} C_{s}=\left(4\int I\left(V\right)dV\right)/Sv\Delta V \end{array}$$

***Cs***(mF/cm^2^) is the specific area capacitance of the electrode, **∫*I (V)dV***is the integral area of CV curves, ***S***(cm^2^) is the actual charging and discharging area of the electrode, ***v***(mV/s) is the scan rate, and **Δ*V***(V) is voltage window. The specific area capacitance of quasi-solid-state flexible SCs can be calculated according to the equation:

(2) $$\begin{array}{l} C_{s}=\left(\int I\left(V\right)dV\right)/Sv\Delta V \end{array}$$

## Results and Discussion

**Figure 1:**
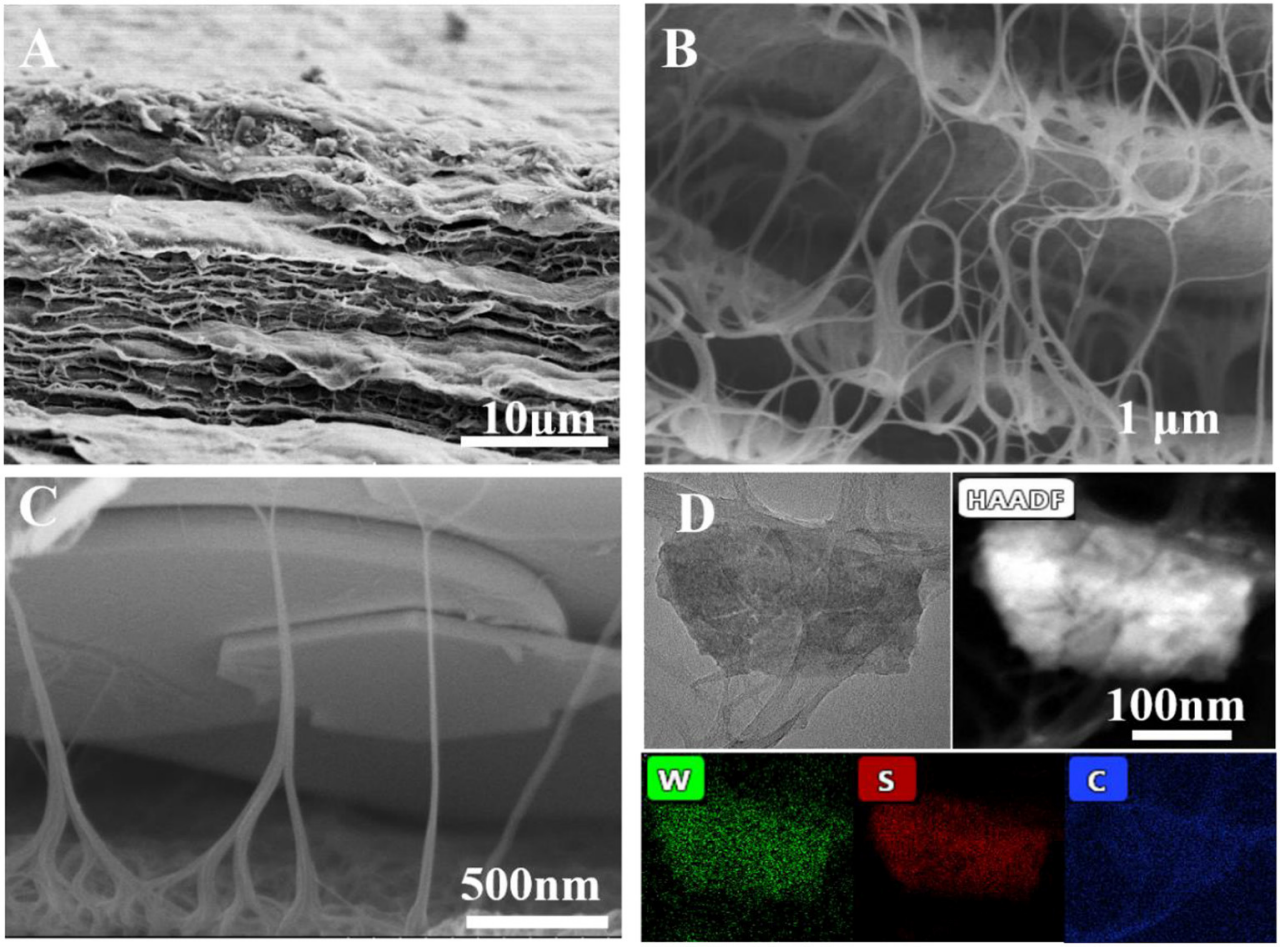
**(A–C)** Cross-section SEM images of WS_2_@CNTs thin-film hybrid with unique skeleton structure at different magnifications. **(D)** The STEM BF image of the WS_2_@CNTs thin-film hybrid electrode and the STEM-EDS maps show the spatial distribution of W, S, and C elements.

TEM and SEM were used to characterize the morphologies of pure WS_2_ nanosheets and the WS_2_@CNT hybrid film. HRTEM images demonstrate the lattice fringes of the WS_2_ nanosheets; through comparison, it can be seen that lithium intercalation can effectively exfoliate the bulk WS_2_ (Figure S1). Figures 1A–C are the SEM images of the WS_2_@CNT hybrid film. The fracture surface of the film presents an obvious and dense layered structure (Figure 1A). In addition, as the enlarged SEM images in Figures 1B,C show, some of the CNTs introduced coat the surface of the WS_2_ nanosheets, and a few of them form a bridge to connect the layers. There are two keys to forming the stack-proof skeleton structure. First of all, CNT, being a light one-dimensional material, is easier to align with the direction of water flow at a certain flow rate. The other key to maintaining this structure is to freeze and finalize the hybrid film in time before it is completely drained; that is, while there is a certain amount of water. The formation and maintenance of the unique structure determine whether the CNTs can be used as a skeleton to support the heavier WS_2_ nanosheets. This particular structure reduces the stacking of the nanosheets and simultaneously increases the interlayer space and ion transfer channels, which is beneficial to electrolyte infiltration and barrier transfer, afterwards providing a larger number of active sites for fast and reversible surface redox reactions. The elemental mapping analysis by energy-dispersive X-ray spectroscopy (EDS) for a WS_2_@CNT thin-film hybrid electrode reveals the uniform distribution of W, S, and C (Figure 1D). In contrast to the hybrid film, which has the unique skeleton structure obtained by freeze-drying, we found that, on naturally dried films obtained at room temperature, the CNTs completely coat the surface of the WS_2_ nanosheets; there is no connecting structure between the layers (Figure S2). It is known to all that the stack-proof skeleton structure and higher specific surface area are key factors in achieving better capacitance characteristics for electrodes, and the BET results show that the freeze-dried WS_2_@CNT thin-film hybrid with a unique skeleton structure displayed 73.13 m^2^/g of surface area, which is higher than that of naturally dried film (48.21 m^2^/g). In order to choose a suitable electrolyte, firstly, the electrochemical performance of freeze-dried film electrodes with a ratio of WS_2_ to CNTs of 1:1 was tested under the acid (1M H_2_SO_4_), neutral (1M Na_2_SO_4_), and alkaline (1M NaOH) conditions. The influence of acid-base on the electrochemical performance of the hybrid film electrode was not significant, and the CV curves showed a larger area in sulfuric acid, so 1M H_2_SO_4_ was chosen as the electrolyte (Figure S3A). As expected, when we compared the electrochemical properties of the films obtained by the two different drying methods, the CV curve of the freeze-dried film revealed a larger scanning area at a scan rate of 20 mV/s (Figure S3B). This further proves that different microstructures have a significant influence on the properties of the films, among which, higher specific surface area and a stack-proof skeleton structure are important factors for improving the electrochemical properties of the hybrid film.

**Figure 2:**
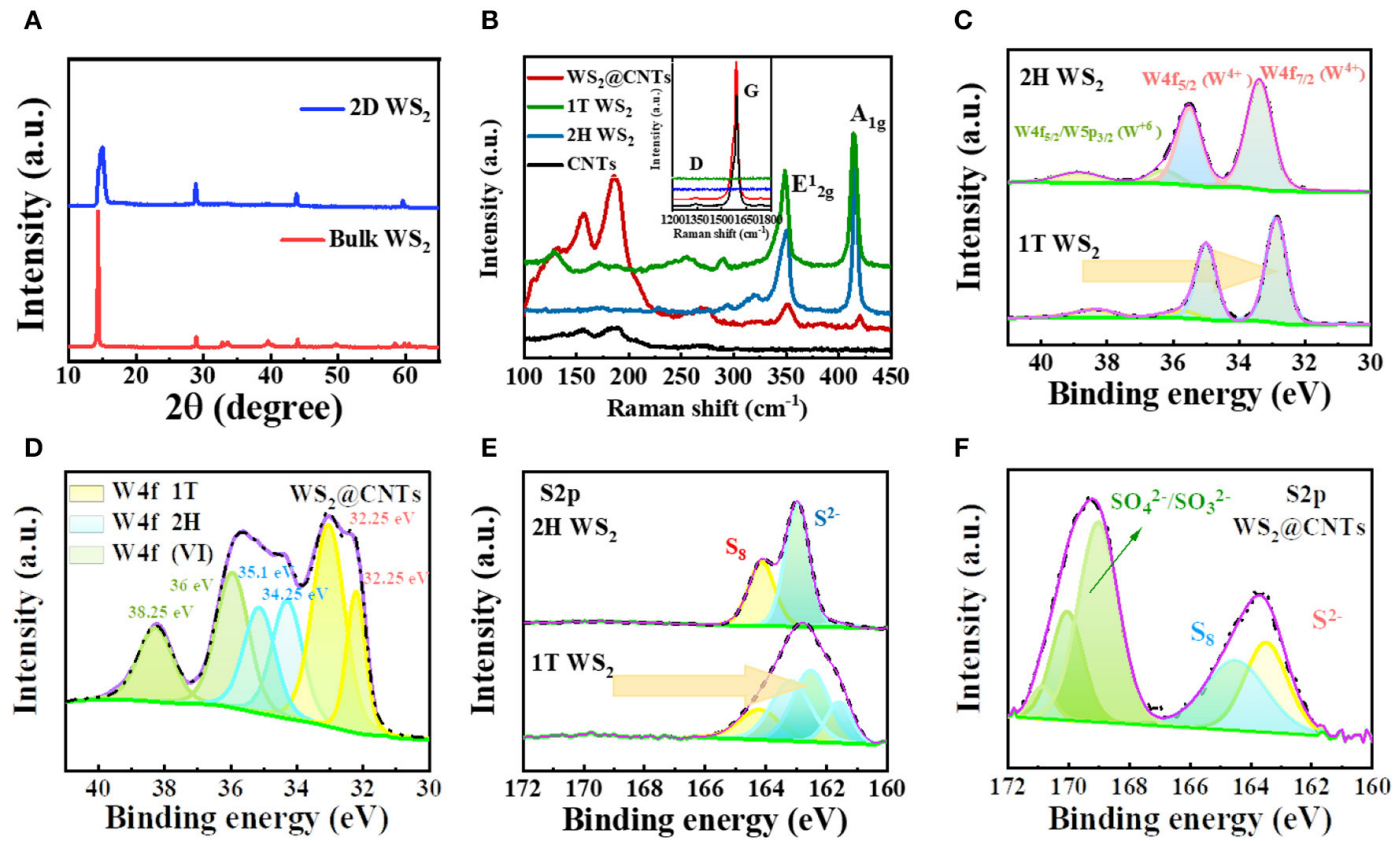
**(A)** XRD patterns of bulk WS_2_ and 2D WS_2_. **(B)** Comparison of Raman results for bulk WS_2_, 2D WS_2_, CNTs, and WS_2_@CNT hybrid films. **(C)** XPS results from the W4f region for 2H–WS_2_, 1T-WS_2_, and **(D)** WS_2_@CNT hybrid film. **(E)** XPS results from the S2p region for 2H-WS_2_, 1T-WS_2_, and **(F)** WS_2_@CNT hybrid film.

The crystal structures of the commercial bulk WS_2_ and the resultant 2D WS_2_ were evaluated by XRD and XPS. As can be seen in the XRD spectra in Figure 2A, all the peaks are well indexed to the 2H bulk phase WS_2_ (PDF#08-0237). After liquid exfoliation treatment, the predominant diffraction peak (2θ=14.3°) becomes wider, and characteristic peaks at 32.8° (100), 33.6° (101), and 39.5° (103) almost disappear, which indicates that there is a phase transformation from 2H to 1T phase. The weakness and disappearance of predominant peaks demonstrate a significant decrease in the degree of crystallinity of WS_2_. XPS tests of raw and liquid exfoliated WS_2_ were further applied to prove this result. For the 2H-WS_2_, in the high-resolution spectrum of the W4f region, the peaks at 33.35 and 35.45 eV are in good accordance with W4f_7/2_ and W4f_5/2_. It is observed that the components of the 1T phase in the high-resolution W4f spectrum are 0.7 eV lower than those belonging to the 2H phase (Figure 2C). This observation is also confirmed by the S2p_3/2−1/2_ spectrum in Figure 2E, where a single peak corresponds to the S^2−^ in 2H-WS_2_ (163.05 eV), and the three changed peaks are attributed to S^2−^ in 1T-WS_2_ (161.55, 162.5, 163.2 eV). These peak shifts also suggest local phase transition from 2H phase to 1T phase.

The Raman spectra of bulk WS_2_, 2D WS_2_, CNTs, and WS_2_@CNT hybrid films are displayed in Figure 2B. The fingerprints below 500 cm^−1^ represent two vibration modes of WS_2_, the out-of-plane A_1g_ and in-plane ${\text{E}}_{2\text{g}}^{1}$ modes. There are two strong-featured peaks for 2H-WS_2_ located at 351 cm^−1^ (A_1g_) and 417 cm^−1^ (${\text{E}}_{2\text{g}}^{1}$), respectively, and for 1T-WS_2_, these two feature peaks slightly shift to 348.8 cm^−1^ (A_1g_) and 413.8 cm^−1^(${\text{E}}_{2\text{g}}^{1}$). This can be explained in terms of changes in the number of WS_2_ nanosheets. In addition, several new peaks appear in the low wavenumber region for 1T-WS_2_ samples. As to the WS_2_@CNT hybrid film, the ratio of ${\text{E}}_{2\text{g}}^{1}$/A_1g_ increases significantly, and, besides the feature peaks of 1T-WS_2_, it shows a strong peak located at 1589 cm^−1^ and a tiny peak located at 1347 cm^−1^, which could be attributed to the G band and D band of CNTs (the D is related to the defective nature of CNTs, and the G peak is associated with the planar C-C stretching vibrations in a crystalline graphitic material). Furthermore, XPS was conducted to identify the chemical composition of the WS_2_@CNTs samples; the spectrum is shown in Figure 2D. It is apparent that W4f peaks are located at various characteristic binding energies in the integral spectrum of WS_2_@CNT films, which implies that besides 1T-WS_2_, a certain amount of 2H-WS_2_ polymorph and partially oxidized tungsten compound coexist in the hybrid system. The presence of 2H-WS_2_ can be explained by the restacking of some nanosheets due to the presence of high pressure during the filtration process. The reason for the presence of oxidized tungsten is that part of the WS_2_ was oxidized during the exfoliation process. The absorption peak at 163.5 eV of S2p proves the existence of S^2−^ in the WS_2_@CNT hybrid film (Figure 2F). The other peaks (~170 eV) were assigned to sulfate (${\text{SO}}_{4}^{2-}$) and sulfite (${\text{SO}}_{3}^{2-}$), which are likely produced through the oxidation of sulfuric acid during the integration process of WS_2_ and CNTs.

**Figure 3:**
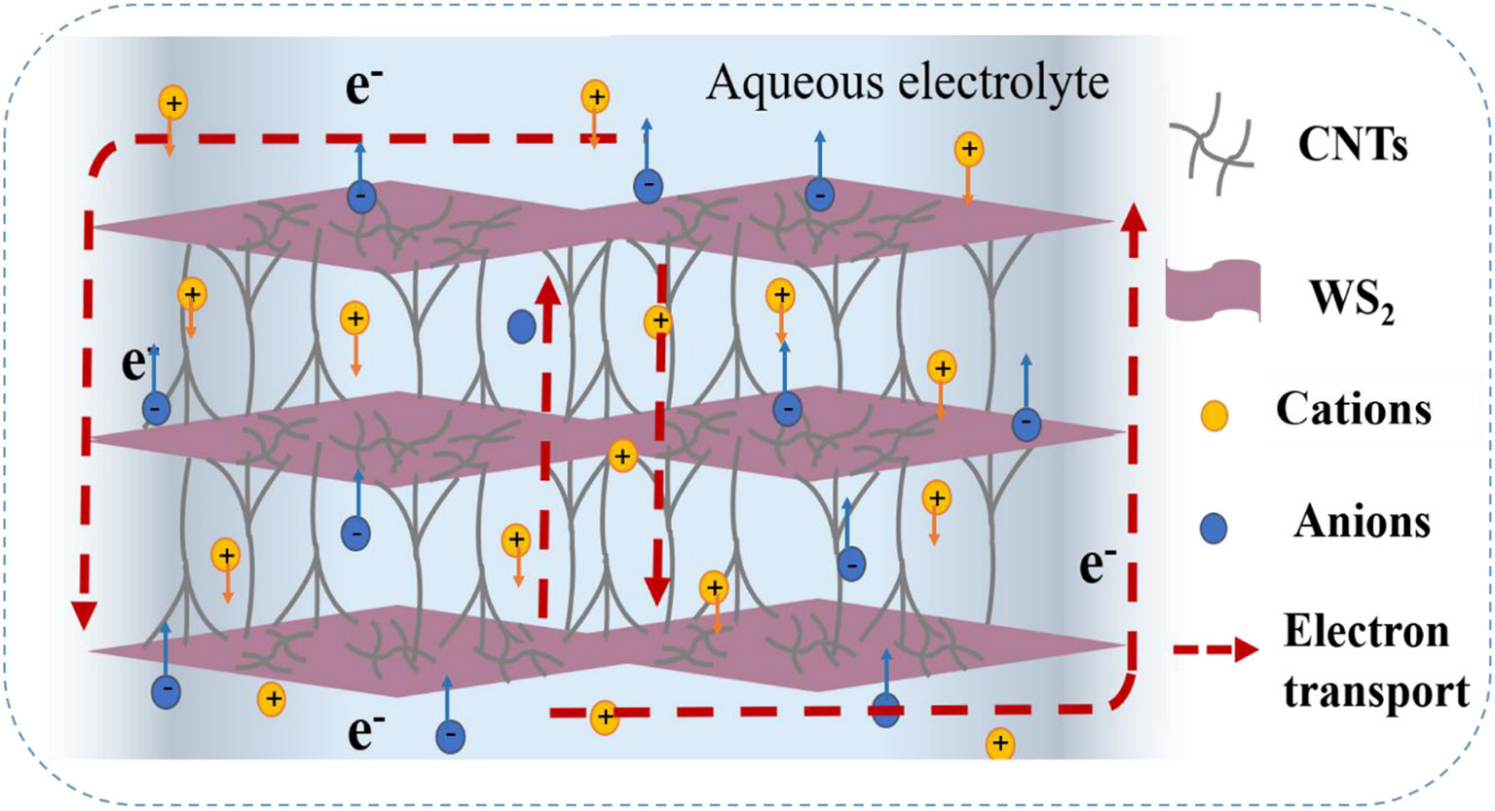
Schematic illustration for the WS_2_@CNT thin-film hybrid with a unique skeleton structure.

The unique nanostructure of WS_2_@CNT hybrid films enables excellent performance when they are used as high-capacity SC electrodes. As shown in Figure 3, WS_2_ nanosheets obtain a larger amount of interlamellar space under the support of CNTs, which offers fast transmission pathways for electrons between the layers, and on the other hand, the loose WS_2_ nanosheets are conducive to deep and unobstructed diffusion for ions throughout the interlayer gaps. By coating the surface of WS_2_, the CNTs also tend to increase the active specific surface area of the WS_2_@CNT hybrid film, thus improving conductivity and facilitating ion transport. In addition, the WS_2_@CNT hybrid films exhibit hydrophilic properties, good mechanical strength, flexibility, and decent electrochemical performance. For all these reasons, the use of this free-standing film as an electrode has great potential for all-solid flexible SCs (Figure S4).

To determine the optimal ratio of CNTs to WS_2_ nanosheets, we tested the electrochemical performance of WS_2_@CNT hybrid films with different proportions of the two materials (CNTs:WS_2_ = 1:6~5:1). With an increasing proportion of CNTs in the hybrid structure, the capacitive contribution of WS_2_ nanosheets is gradually overwhelmed, and hence the capacitive property of the hybrid films is close to that of pure CNTs. This is mainly due to the fact that the density of the CNTs is smaller than that of WS_2_, so the volume of CNTs is much larger than that of WS_2_ for the same mass. In the hybrid film, when the mass fraction of CNTs is too large, WS_2_ will be covered by a large number of CNTs, eliminating the stack-proof skeleton structure, and thus causing the advantage of the unique microstructure to be negligible (Figure S5). By comparison, we find that when CNTs:WS_2_ is 1:5, the CV curve possesses the largest area (Figure S6).

**Figure 4:**
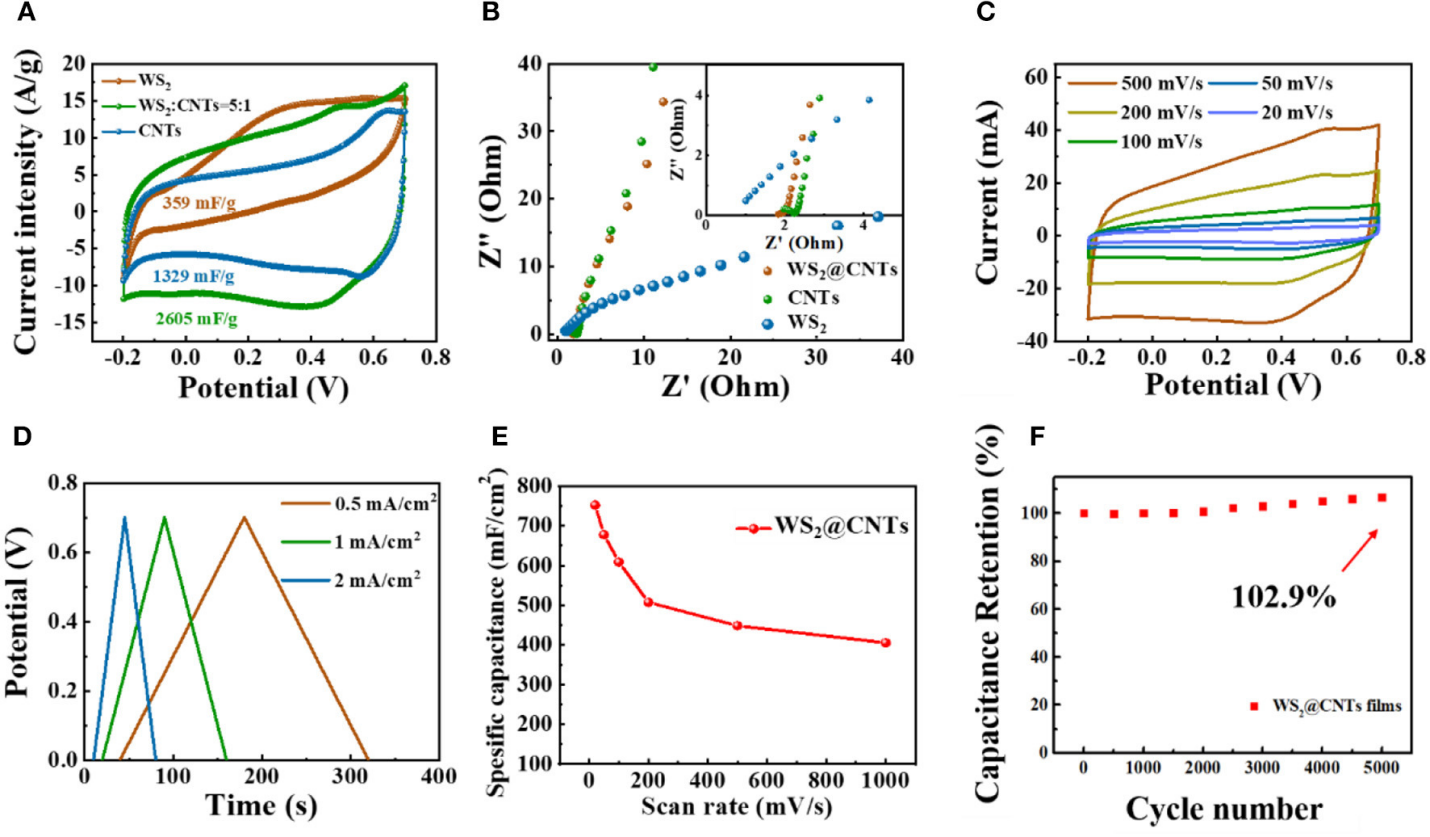
Electrochemical performances of the WS_2_@CNT hybrid films tested in a three-electrode system. **(A)** Typical CV curves (scan rate of 50 mV/s) and **(B)** EIS curves of pure CNTs, WS_2+_, and WS_2_@CNT hybrid films. **(C)** CV curves of WS_2_@CNT hybrid films under scan rates from 20 to 500 mV/s. **(D)** GCD curves measured at different current densities. **(E)** Rate performance of hybrid film under scan rates from 20 to 1000 mV/s and **(F)** cycling stability measured at 100 mV/ s for 5,000 cycles.

To demonstrate the superiority of the bridging structure of WS_2_@CNT hybrid film (100 mL solution with a CNTs to WS_2_ ratio of 1:5) and further explore the contribution of different components to the capacitance, CV and electrochemical impedance spectroscopy (EIS) measurements were obtained for WS_2_@CNT hybrid film, pure CNTs, and WS_2_ films with a three-electrode configuration. Figure 4A shows CV curves at a scan rate of 50 mV/s, and the corresponding capacitance is calculated, respectively. The reason we use mass-specific capacitance here rather than area- or volume-specific capacitance is because mass-specific capacitance is more convincing for comparison with the electrochemical properties of the material itself. By comparison, it can be found that the CV curve of WS_2_ is irregular and shows a spindle shape, which is attributed to its poor electrical conductivity. Although WS_2_ has capacitance characteristics, the mass-specific capacitance is only 359 mF/g; this is mainly due to the common problem of easy stacking of 2D materials, which reduces the contact area between active substances and electrolytes. CNTs have good conductivity, and the introduction of a small amount of carbon nanotubes into the hybrid film can effectively enhance its conductivity. At the same time, the insertion of CNTs between the WS_2_ nanosheets also effectively prevents stacking. We find that the CV curve of WS_2_@CNT hybrid film exhibits more obvious capacitive characteristics together with a capacitance that is than the others and higher than the simple superposition of the mass-specific capacitance values of pure CNTs and WS_2_. Therefore, in hybrid films, CNTs exist as stack-proof skeletons that enhance conductivity, while WS_2_ provides a more active substance; this two-component synergy can increase the contact area between active substances and electrolytes and increase the accessibility of active sites. In the impedance spectra (Figure 4B), the slope at low frequency concerning the hybrid film is much higher than that of the sample without CNTs; this results from the increases of conductivity and layer spacing after introducing CNTs into the interlayer region of WS_2_ nanosheets, which is instrumental in ion transmission and electrolyte infiltration. Consequently, the diffusion resistance between layers is well-reduced.

In order to testify to the electrochemical performance of the WS_2_@CNT hybrid film, the CV curve measurements were performed in 1 M H_2_SO_4_ solution under various scan rates from 20 to 500 mV/s (Figure 4C). All of the CV curves of the film present nearly symmetrical rectangular shapes, indicating exceptional stability and potentials even under a high scan rate of 500 mV/s. Figure 4D shows GCD curves of the WS_2_@CNT hybrid film at different current densities, respectively. The curves are predominantly linear, without an obvious IR drop at the start of each discharge curve. This linearity of the discharge curve is proof of good capacitive behavior and nearly ideal charge/discharge performance. For the film electrodes, the weight and thickness are too negligible to use the performance metrics of gravimetric- and volumetric-specific capacitances and energy density, and miniaturized energy storage devices should be considered for their effective footprint area rather than their weight. Therefore, here the specific areal capacitance, energy, and power density are used as the standard performance metrics for our WS_2_@CNT hybrid film electrode and quasi-solid-state flexible SCs. The specific areal capacitance of the electrode based on WS_2_@CNT hybrid film can be calculated from the CV curves according to Equation (1). Figure 4E plots the calculated specific capacitance values with an increase in scan rate from 20 to 500 mV/s. There is a decrease in specific capacitance with increasing scan rate due to the transport limit on the supply of ions. The specific areal capacitance was measured to be 752.53 mF/cm^2^ at 20mV/s, decreasing to 405.22 mF/cm^2^ with capacitance retention of ~53.85% at 500 mV/s. The WS_2_@CNT hybrid film shows a ~2.9% increase in capacitance (Figure 4F) after 5,000 cycles. This increased capacitance with continuous cycling is attributed to the partial exfoliation with continuous ion intercalation/deintercalation increasing the active surface area, termed “electroactivation” (Lorenzo and Srinivasan, 2018). This indicates that a composite of both WS_2_ and CNTs together is better able to resist structural changes that occur with continual charge and discharge cycles.

**Figure 5:**
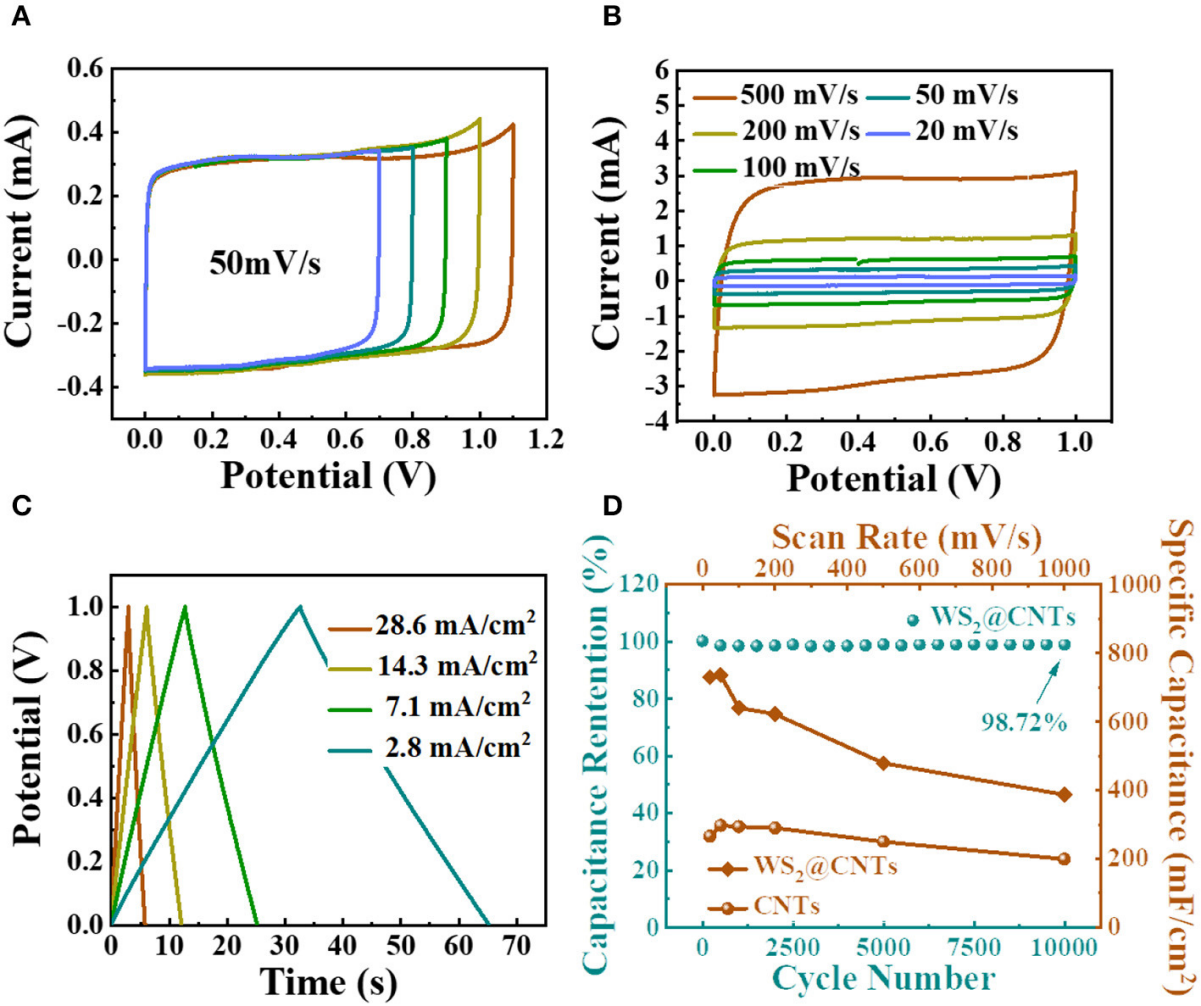
Electrochemical performances of the WS_2_@CNT hybrid films tested in a two-electrode system. **(A)** CV curves of WS_2_@CNT hybrid films in various voltage windows at a scan rate of 50 mV/s. **(B)** CV curves of WS_2_@CNT hybrid films at scan rates from 20 to 500 mV/s. **(C)** GCD curves measured at different current densities. **(D)** Rate performance of WS_2_@CNT hybrid film and CNT film at scan rates from 20 to 1000 mV/s, together with cycling stability measured at 100 mV/ s for 10, 000 cycles.

To further estimate the electrochemical performance of the WS_2_@CNT hybrid films, capacitance measurements were carried out in a two-electrode Swagelok cell configuration with 1 M H_2_SO_4_. In this system, glassy carbon electrodes were used as current collectors. In Figure 5A, we explored potentials with regard to the electrochemical windows of the hybrid films. As seen from the spectra, the CV curves maintain a relatively symmetrical rectangular shape, and there is no obvious fluctuation until the voltage window is enlarged to 1.0 V. Then the WS_2_@CNT hybrid films present typical capacitive behavior, with a perfect rectangular shape of the CV curve, and it remains normal even at a scan rate of 500 mV/s (Figure 5B). At this scan rate, the current can respond to the change in voltage quickly, which indicates that electrolyte can diffuse rapidly through the film. This is also related to the smaller gap between the two electrodes in the Swagelok cell structure. Similarly, GCD curves at various current densities were investigated in a stable voltage window range from 0.0 to 1.0 V (Figure 5C). The linear time-potential profiles and the symmetrical characteristics during the charge/discharge process, without obvious IR drop, represent ideal capacitive properties, with a rapid I-V response and small equivalent series resistance, which are also in agreement with the CV curves in Figure 5B. As shown in Figure 5D, to start with, the specific capacitance of the WS_2_@CNT hybrid film is much higher than that of pure CNTs. The maximum specific capacitance of WS_2_@CNT hybrid film reaches 744 mF/cm^2^, which is basically consistent with the measured data in a three-electrode system (752.53 mF/cm^2^), significantly surpassing the specific capacitance of CNTs (297.6 mF/cm^2^). Otherwise, the hybrid film shows better rate performance in comparison to pure CNT film. The capacitance retention of hybrid film is maintained at 98.72% even after 10,000 cycles, signifying ideal capacitive behavior and exceptional cycling stability during a long lifespan in use.

**Figure 6:**
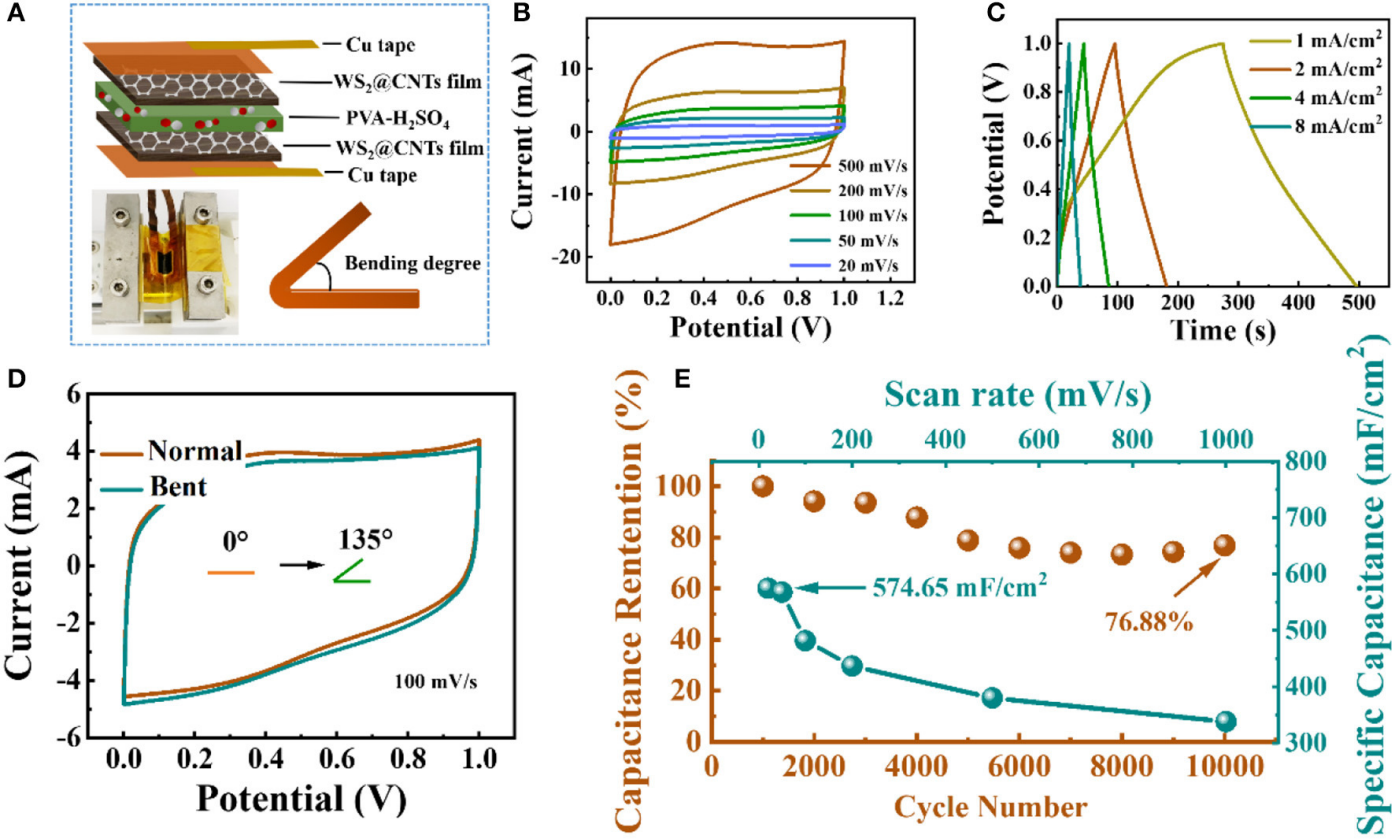
Electrochemical performances of the quasi-solid-state flexible SCs based on WS_2_@CNT hybrid films. **(A)** Schematic illustration and digital photographs of the fabricated quasi-solid-state flexible SCs. **(B)** CV curves of quasi-solid-state flexible SCs from 20 to 500 mV/s. **(C)** GCD curves measured at different current densities. **(D)** CV curves of quasi-solid-state flexible SCs tested under different degrees of bending at a scan rate of 100 mV/s, respectively. **(E)** Scan rate stability test of quasi-solid-state flexible SCs from 20 to 1000 mV/s and cycle stability measured at 100 mV/s for 10,000 cycles.

For the sake of solving the problem of leakage of liquid electrolyte in conventional SCs, we encapsulated the quasi-solid-state flexible SCs using WS_2_@CNT hybrid films as the active electrode materials with a solid PVA/H_2_SO_4_ electrolyte (Figure 6A). The CV curves of the quasi-solid-state flexible SCs under different scan rates are given in Figure 6B. The CV curves all present a normal rectangular profile within a potential range from 0 to 1.0 V, and this condition remains steady even at a high scan rate of 500 mV/s, indicating rapid current response and fast diffusion of ions in the electrodes. Voltage vs. time profiles were obtained by applying the galvanostatic charge/discharge technique at different currents. As shown in Figure 6C, the plot shows highly linear behavior and a symmetrical shape, which also signifies an ideal capacitive behavior for the quasi-solid-state flexible SCs. Additionally, inspection of the CV curves in Figure 6D indicates that when bending at 135 degrees, the quasi-solid-state SC still delivers a rectangular CV profile that is coincident with the normal state, without obvious capacitance loss or electrochemical failure, which indicates the feasible flexibility and stability of the device. In order to further verify the importance of the stack-proof structure to the electrochemical performance, we took the edge of the bent part of the hybrid film electrode after the quasi-solid-state SC had been bent 5,000 times in the collider, observed its micro-morphology by SEM, and compared its CV curve with that of the unbending part. It was found that the area of the CV curve of the electrode with a damaged structure is much smaller than that of an electrode with a stack-proof skeleton structure, which indicated that the stack-proof skeleton structure is the key to maintaining the high performance of the hybrid film electrode (Figure S7). As presented in Figure 6E, the areal specific capacitance of a quasi-solid-state flexible SC assembled with a WS_2_@CNT hybrid electrode could reach 574.65 mF/cm^2^. When the scanning rate reaches 1000 mV/s, the areal specific capacitance still has about 60% retention. After 10,000 cycles of half-length bending, the specific capacitance of the quasi-solid-state flexible SC only decreases by ~23% from its maximum capacitance, meaning that it has decent cycling stability when it comes to complicated use circumstances in practice. To go a step further, the energy density (***E***, mWh/cm^2^) and the power density (***P***,mW/cm^2^) of devices can be calculated using the following equations:

(3) $$\begin{array}{lll} E & = & \left(\frac{1}{2}C\Delta V^{2}\right)/3600 \end{array}$$

(4) $$\begin{array}{lll} P & = & E/\Delta t\times 3600 \end{array}$$

where ***C***(mF/cm^2^) is the specific capacitance, calculated from Equation (2), **Δ*V***is the sweep potential window, and **Δ*t*** is the discharge time (s). This quasi-solid-state flexible SC delivers an energy density of 0.0798 mWh/cm^2^ at a power density of 5.745 mW/cm^2^. A comparison for the electrochemical performance of 2D TMD-based supercapacitors is summarized in Table S1 (Wang et al., 2009; Mayorga-Martinez et al., 2016; Shang et al., 2017; Susarla et al., 2017; Yang et al., 2017; Lin et al., 2018; Gupta et al., 2019; Tolar, 2019). By contrast, it can be found that the specific capacitance, energy density, and power density obtained in this work reach a high standard. These outstanding performances arise not only from the unique hybrid nanocomposite structure of the electrode material but also from the introduction and bridge-bonding of CNTs to enlarge the layer spacing.

## Conclusion

In conclusion, a skeleton-structured WS_2_@CNT thin-film hybrid electrode with good mechanical properties and electrochemical performance was prepared by a facile self-assembly method combining vacuum filtration and freeze-drying. This simple and easy-to-operate preparation method is also applicable for the preparation for other 2D TMD@CNT thin-film electrodes besides WS_2_. The hybrid film electrode presented the best electrochemical performance when CNTs and WS_2_ were combined at a mass ratio of 1:5. The area-specific capacitance could reach up to 752.53 mF/cm^2^, and it retained 98.72% of the initial capacitance after 10,000 cycles. Based on this hybrid electrode, a quasi-solid-state flexible SC was assembled, and the device exhibited excellent cycling stability under different bending angles, together with a 574.65 mF/cm^2^ area-specific capacitance and decent capacitance retention of 76.88% after 10,000 bending cycles, which demonstrated that this hybrid film electrode and associated energy-storage device have great application potential in the field of energy management for flexible and lightweight electronics.

## Data Availability Statement

All datasets presented in this study are included in the article/Supplementary Material.

## Author Contributions

XY and JL contributed the conception and design of the study. XY wrote the draft of the manuscript. All of the authors contributed to manuscript revision and read and approved the submitted version.

## Conflict of Interest

The authors declare that the research was conducted in the absence of any commercial or financial relationships that could be construed as a potential conflict of interest.
