# Supplementary material for: Skeleton-Structure WS2@CNT Thin-Film Hybrid Electrodes for High-Performance Quasi-Solid-State Flexible Supercapacitors
Source: Front Chem. 2020 Jun 12;8:442. doi: 10.3389/fchem.2020.00442 (PMC7303003; doi:10.3389/fchem.2020.00442)
Supplement: Supplementary file 1 [file Table_1.DOCX]

Supplementary Material


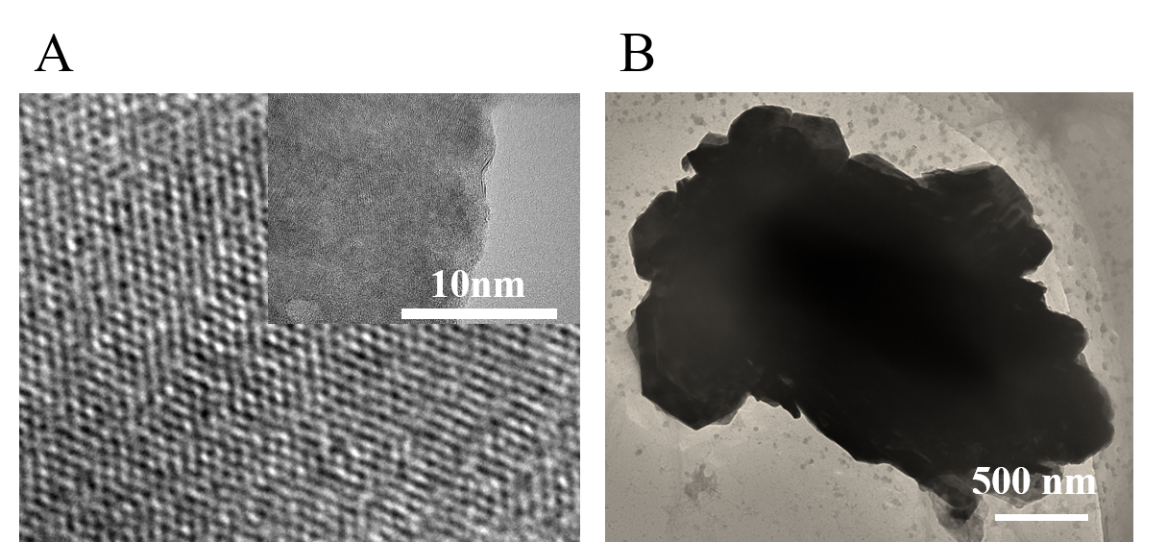


**Figure S1** (A) HR-TEM image of the WS_2_ nanosheets at different magnifications. (B) TEM image of the bulk WS_2_.


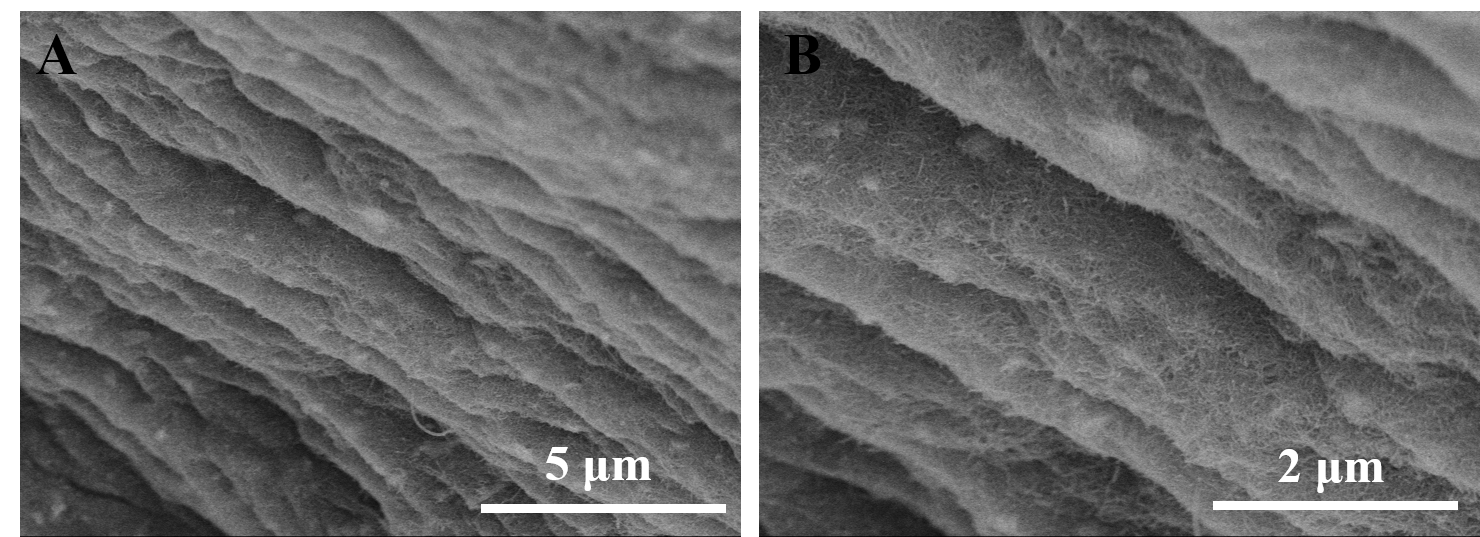


**Figure S2** SEM images of naturally-dried WS_2_@CNTs film at room temperature.


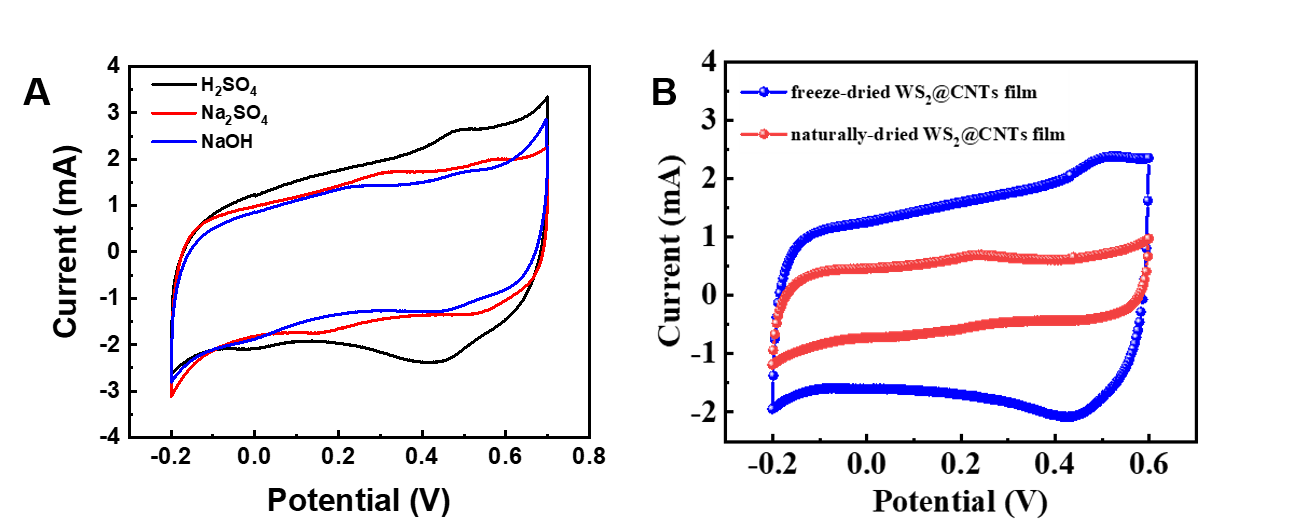


**Figure S3** (A) Comparison of CV curves of WS_2_@CNTs films in different electrolytes at a scan rate of 20 mV/s. (B) Comparison of CV curves of WS_2_@CNTs films obtained by different drying methods.


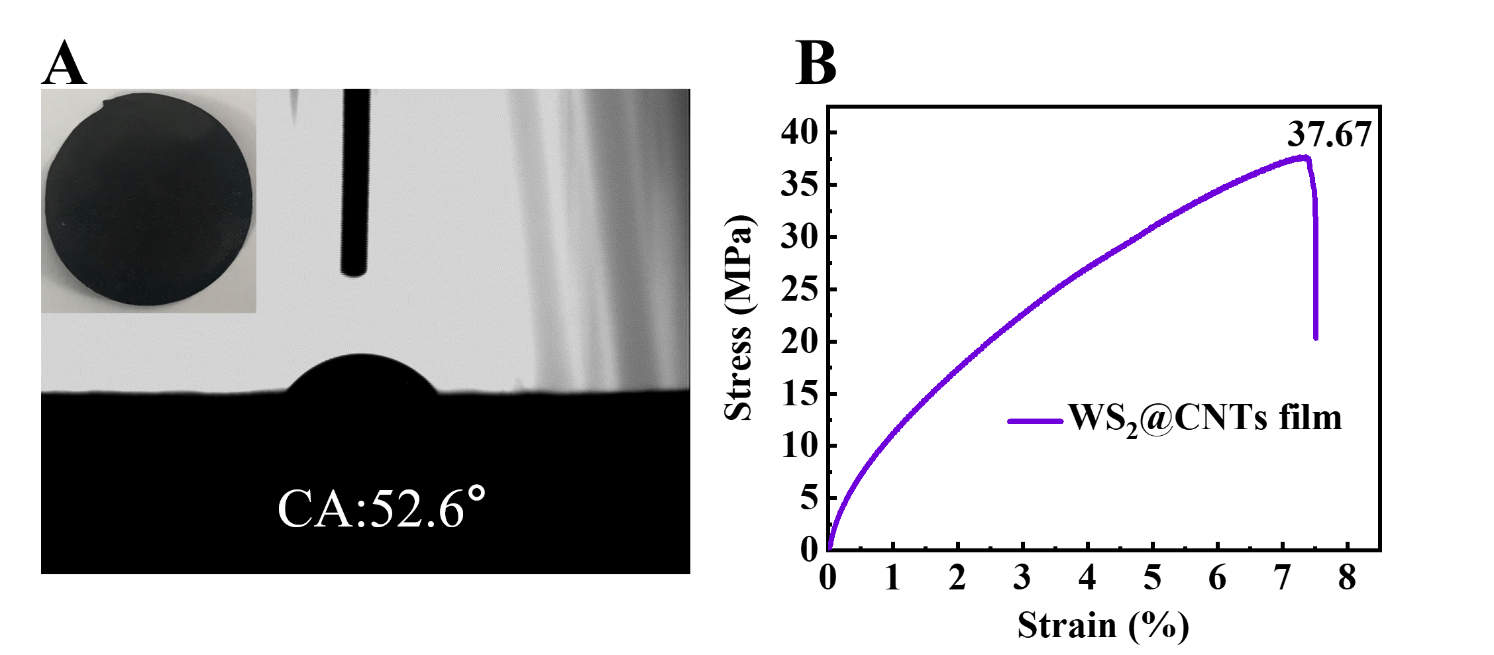


**Figure S4** (A) Hydrophobic test and (B) the plot of strain versus stress of WS_2_@CNTs film.


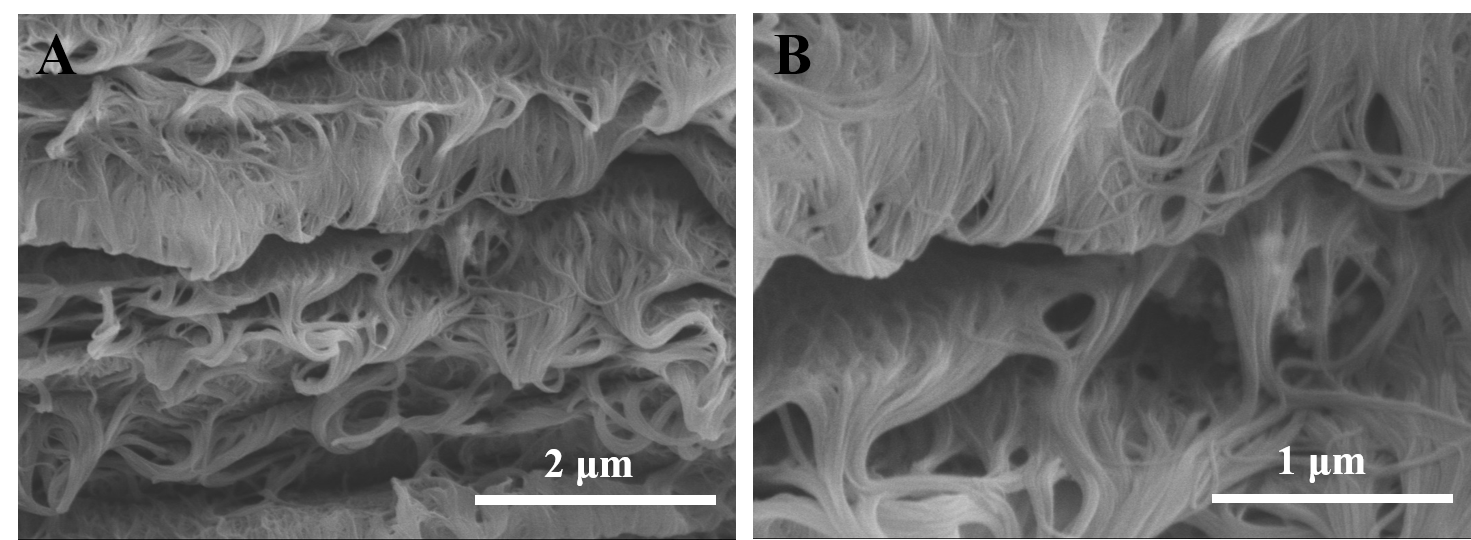


**Figure S5** SEM images of WS_2_@CNTs films with the mass ratio of 5:1 for CNTs to WS_2_.


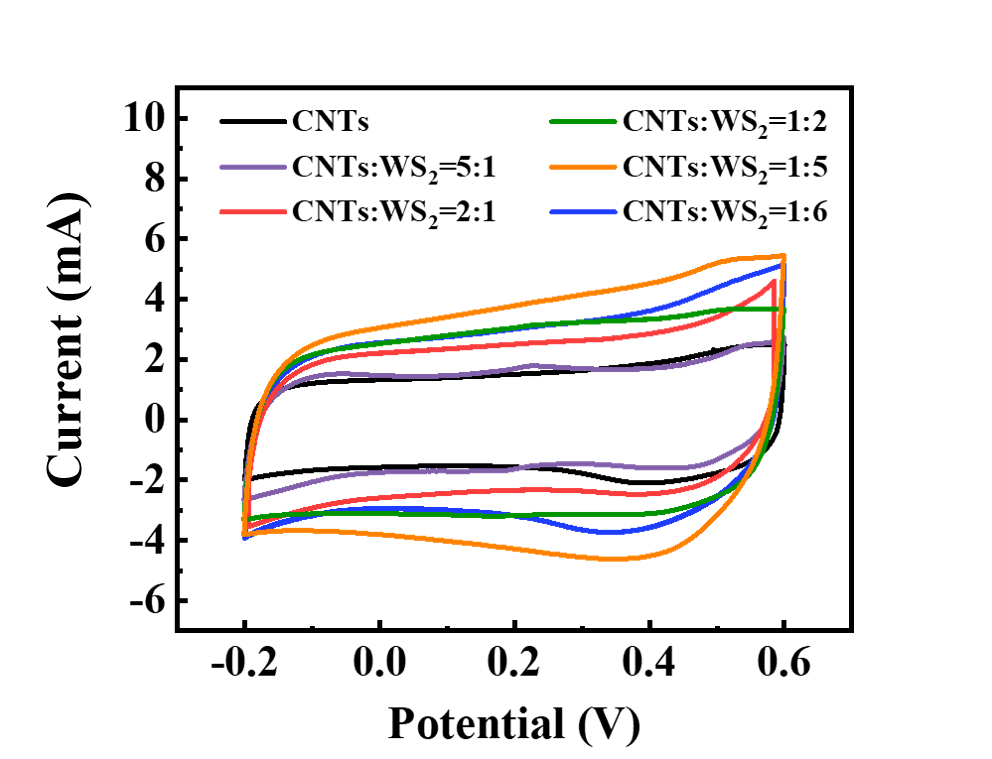


**Figure S6** Comparison of CV curves between CNTs film and WS_2_@CNTs films doped with different proportion of CNTs and WS_2_ nanosheets (mass ratio CNTs:WS_2_= 5:1~1:6).

.


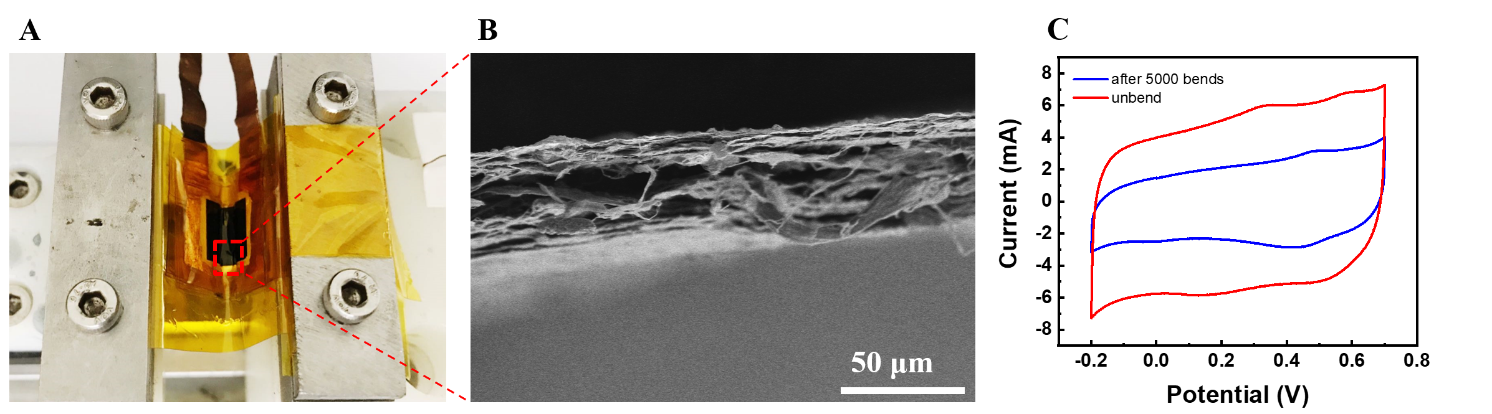


**Figure S7** (A) Digital photo of quasi-solid-state flexible SCs in bending state. (B) SEM photo of hybrid film electrode after 5000 bands. (C) Comparison of CV curves between bent 5000 times and unbend hybrid film electrodes.

**Table S1** Comparison for electrochemical performance of 2D materials-based supercapacitors.

| Materials | Electrolyte | Capacitance of device | Energy density | Power density | Resistance (Cycles) | Bending (Cycles) | Ref |
| --- | --- | --- | --- | --- | --- | --- | --- |
| MoS_2_@rGO‐CNT |  | 13.7mF/cm^2^  40 F/cm^3^ | 1.9 µWh/cm^2^  5.6 mWh/cm^3^ |  | 96.6%  (10,000 C) |  | 28 |
| 1T-MoS_2_ nanosheets | H_2_SO_4_  (1 M) | 250 F/cm^3^ | 0.016  Wh/cm^3^ | 0.62  W/cm^3^ | 90%  (5000 C) |  | 24 |
| WS_2_ nanoparticles /polyaniline | H_2_SO_4_  (0.5 M) | 1400 μF/cm^2^ | 0.07 mWh/m^2^ | 120  mW/m^2^ | _ |  | 29 |
| AgNWs-MoS_2_ | H_2_SO_4_  (1 M) | 27.6 mF/cm^2^ | 2.453  µWh/cm^2^ | 1.472  mW/cm^2^ | 85.2%  (20,000 C) |  | 29 |
| WS_2_/CFC | KCl  (1 M) | 399 F/g | _ | _ | 99%  (500 C) |  | 27 |
| rGO-MoS_2_-WS_2_ | KOH  (3 M) | 1.7 mF/cm^2^  365 F/g | 15 Wh/kg | 373 W/kg | _ | 70%  (3000 C) | 32 |
| GO | KOH  (30 wt %) | 205 F/g | 28.5 Wh/kg | 10 kW/kg | 90%  (1200 C) | _ | 30 |
| 1T-WS_2_ nanoribbons | H_2_SO_4_  (1 M) | 2813 μF/cm^2^ | _ | _ | 33%  (2000 C) | _ | 23 |
| 2D MoS_2_ | KOH  (3 M) | 255.65 F/g | 35.5 Wh/Kg | 250 W/Kg | 70%  (1000 C) | _ | 31 |
| Our work | H_2_SO_4_  (1 M) | 574.65 mF/cm^2^ | 0.0798  mWh/cm^2^ | 5.745  mW/cm^2^ | 98%  (10,000 C) | 76.88%  (10,000 C) |  |
